# 35 Associations between transport modes and site-specific cancers: A Systematic review and meta-analysis

**DOI:** 10.1093/eurpub/ckae114.148

**Published:** 2024-09-26

**Authors:** Win Thu, Alistair Woodward, Alana Cavadino, Sandar Tin Tin

**Affiliations:** Epidemiology and Biostatistics Unit, University Of Auckland, New Zealand; Epidemiology and Biostatistics Unit, University Of Auckland, New Zealand; Epidemiology and Biostatistics Unit, University Of Auckland, New Zealand; Epidemiology and Biostatistics Unit, University Of Auckland, New Zealand; Cancer Epidemiology Unit, University of Oxford, United Kingdom

## Abstract

**Purpose:**

Transport choices may influence cancer risks through their effects on physical activity levels, sedentary time, and environmental pollution. This review synthesizes existing evidence on the associations of specific transport modes with risks of site-specific cancers.

**Methods:**

Relevant literature was searched up to 17th February 2023 in PubMed, Embase, and Scopus, and results were meta-analyzed for cancer sites where two or more studies were identified.

**Results:**

27 eligible studies (11 cohort, 15 case-control, and 1 case-cohort) were identified, which reported the associations with 10 site-specific cancers (breast, endometrial, colorectal, testicular, prostate, ovarian, lung, renal, liver, gallbladder and biliary tract). In meta-analysis, 10 Metabolic Equivalent of Task hour increment in transport-related physical activity per week (∼ 150 minutes of walking or 90 minutes of cycling for commute) was associated with a 7% reduction in risk for endometrial cancer (95% CI: 0.89 – 0.98), 5% reduction for colorectal cancer (95% CI: 0.91 – 0.99) and 2% reduction for breast cancer (95% CI: 0.89 – 0.996). Compared to motorized modes, cycling was associated with a lower risk of overall cancer incidence and mortality.

**Conclusion:**

Active transport appears to reduce cancer risk, but evidence for cancer sites other than colorectum, breast, and endometrium is currently limited.

